# Evaluation of the Efficacy of OSU-2S in the Treatment of Non-Small-Cell Lung Cancer and Screening of Potential Targets of Action

**DOI:** 10.3390/ph17050582

**Published:** 2024-05-01

**Authors:** Mengyuan Han, Xiangran Liu, Sendaer Hailati, Nurbiya Nurahmat, Dilihuma Dilimulati, Alhar Baishan, Alifeiye Aikebaier, Wenting Zhou

**Affiliations:** 1 Department of Pharmacology, School of Pharmacy, Xinjiang Medical University, Urumqi 830017, China; hanmengyuan@stu.xjmu.edu.cn (M.H.); lxr971220@163.com (X.L.); sendaer@stu.xjmu.edu.cn (S.H.); nurbiye@stu.xjmu.edu.cn (N.N.); dilihuma@stu.xjmu.edu.cn (D.D.); alhar@stu.xjmu.edu.cn (A.B.); alfi110@163.com (A.A.); 2 Xinjiang Key Laboratory of Active Components and Drug Release Technology of Natural Medicines, Urumqi 830017, China

**Keywords:** OSU-2S, NSCLC, tumor models, bioinformatics analysis, Western blot

## Abstract

(1) Background: OSU-2S is a derivative of FTY720 and exhibits significant inhibitory effects on various cancer cells. There is currently no research on the mechanism of the impact of OSU-2S on NSCLC development. We analysed and validated the hub genes and pharmacodynamic effects of OSU-2S to treat NSCLC. (2) Methods: The hub genes of OSU-2S for the treatment of NSCLC were screened in PharmMapper, genecard, and KM Plotter database by survival and expression analysis. The effect of OSU-2S on hub gene expression was verified by Western blot analysis. The ex vivo and in vivo efficacy of OSU-2S on tumour growth was verified using A549 cells and a xenografted animal model. (3) Results: A total of 7 marker genes for OSU-2S treatment of NSCLC were obtained. *AURKA* and *S1PR1* were screened as hub genes. Significant differences in the expression of *AURKA* and *S1PR1* between normal and lung adenocarcinoma (LUAD) tissues were found in the GEPIA2 database; Western blot showed that OSU-2S could affect *p-AURKA* and *S1PR1* protein expression. OSU-2S significantly inhibited tumour growth in A549 cells and xenografted animal models. (4) Conclusions: Our study confirms the inhibitory effect of OSU-2S on NSCLC, screens and demonstrates its potential targets *AURKA*(*p-AURKA*) and *S1PR1*, and provides a research basis for treating NSCLC with OSU-2S.

## 1. Introduction

Lung cancer is the most prevalent cancer globally and kills approximately 350 people each day, which is 2.5 times the number of people who die from rectal cancer. Lung cancer claims the lives of around 130,000 individuals annually all around the world [1]. Small- cell lung cancer (SCLC) and non-small-cell lung cancer (NSCLC) are included in this cancer. Lung adenocarcinoma (LUAD) and squamous lung cancer (LUSC) are the two histologic subtypes of NSCLC, which is more common in comparison to SCLC [2]. Lung adenocarcinoma is the most prevalent, and its incidence rate accounts for approximately 50% of lung cancer cases. The use of targeted agents for treating lung adenocarcinoma has gradually become one of the most popular therapeutic approaches in the last decade [3,4]. Over 50% of patients with stage I or II non-small-cell lung cancer undergo surgery, in comparison to just 21% of patients with stage III non-small-cell lung cancer, while almost 61% of patients receive chemotherapy and/or radiotherapy [5]. Related studies have found that surgery is the more commonly used treatment for early-stage NSCLC, sometimes combined with other treatments such as chemotherapy, targeted agents, immunotherapy, and/or radiation therapy. Advanced NSCLC is generally treated with chemotherapy, targeted agents, and/or immunotherapy [6]. As early-stage NSCLC is normally asymptomatic, just 30% of cases are diagnosed during the first stage and the majority of cases are in advanced stages by the time of diagnosis [7]. Therefore, drug therapy for advanced NSCLC is essential. The potential targets and related mechanisms of new targeted anti-tumour drugs for lung adenocarcinoma treatment urgently require basic research.

**Figure 1:**
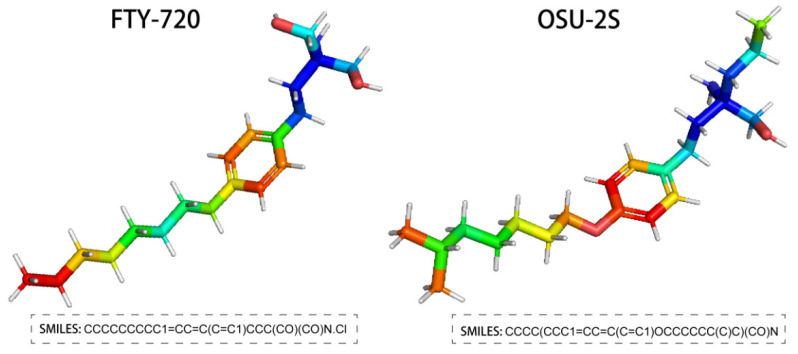
Structure of FTY720 and OSU-2S.

FTY720, the generic name Fingolimod, is an FDA-approved sphingosine immunosuppressant that targets the sphingosine-1-phosphate (S1P) receptor for treating relapsing multiple sclerosis. It is extracted from Cordyceps [8,9]. In addition, FTY720 has been reported to have anti-tumour effects on a range of cancer types, including breast, bladder, liver, and ovarian cancers [10]. According to related research, FTY720 activates the ROS/PKCδ-signalling pathway in hepatocellular carcinoma cells, which in turn mediates the inactivation of caspase-3 [11,12]. To avoid the immunosuppressive effect of FTY720, a non-immunosuppressive FTY720 derivative OSU-2S was created by structural modification [13,14]. In comparison to FTY720, OSU-2S maintained FTY720’s capacity to increase PP2A activity and cause PKCδ breakage and caspase-dependent cell death [15]. This provides a mechanistic basis for OSU-2S to become an excellent anti-tumour drug. The structure of FTY720 and OSU-2S is shown in Figure 1.

The specific mechanism of the action of the new non-immunosuppressive antitumour drug OSU-2S is not very clear. Previous relevant in vitro and in vivo studies have found OSU-2S to have obvious cytotoxicity in acute lymphocytic leukaemia (ALL), chronic lymphocytic leukaemia (CLL), mantle cell lymphoma (MCL), and other diseases, as it induces the death of cancer cells [16]. A study of canine spontaneous b-cell lymphoma found that OSU-2S induced reactive oxygen species (ROS) production in canine lymphoma cells, leading to apoptosis [14]. Research on the targets and mechanisms of OSU-2S in anti-tumour treatment requires further study. Therefore, this paper analysed and validated the potential targets of OSU-2S action with NSCLC by utilising network pharmacology and bioinformatics methods.

## 2. Results

### 2.1. Acquisition of Marker Genes

**Figure 2:**
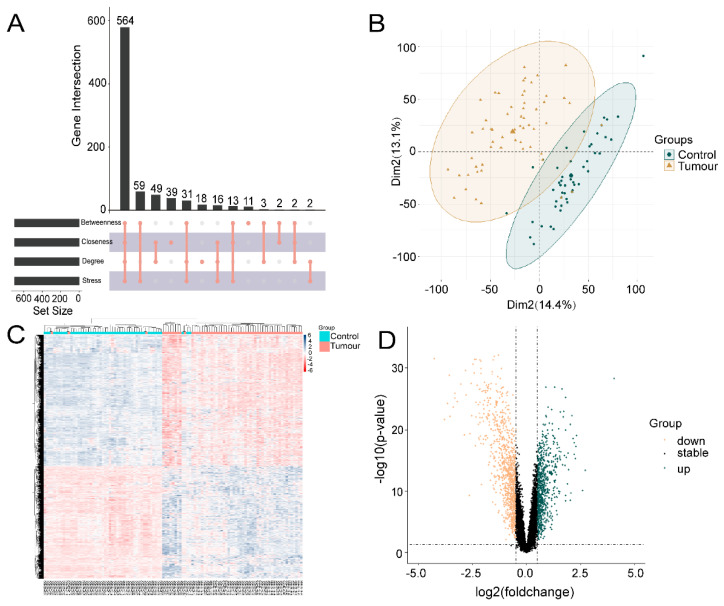
Acquisition of genes associated with lung adenocarcinoma. (**A**) cytoHubba plugin was used to screen for NSCLC disease genes. (**B**) Expression corrected PCA analysis of GSE10072 data matrix, dark green represents normal tissue, light yellow represents tumor tissue. (**C**) Heat map of differentially expressed genes, with red areas showing positive correlation and blue areas showing negative correlation. (**D**) Differentially expressed gene volcano plot, dark green represents up-regulated genes and light yellow represents down-regulated genes.

**Figure 3:**
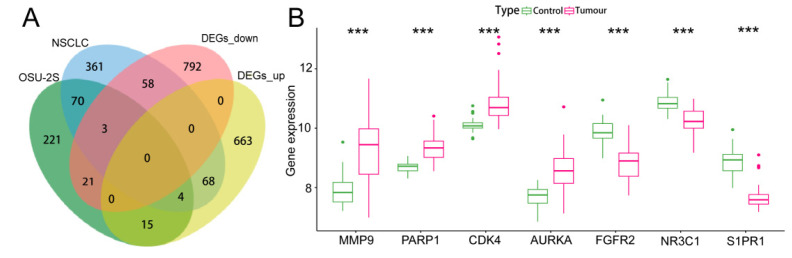
Screening and analysis of marker genes. (**A**,**B**) Venn diagrams of the three intersecting genes, DEGs, OSU-2S targets of action and disease targets, with gene expression box line plots. *** represents *p* < 0.001.

One hundred OSU-2S effector targets were predicted in the Swiss TargetPrediction database, and 299 OSU-2S effector targets were predicted in the PharmMapper database. 334 effector targets remained after the two were combined and duplicates were removed. 1050 and 160 NSCLC targets were obtained from Genecard database and TTD database, respectively. The two were merged to remove duplicate values to obtain 1159 targets, which were analyzed by PPI and uploaded to Cytoscape_v3.7.2 software to screen the top 700 genes by the 4 modules in the cytoHubba plug-in. The intersection was taken in order to obtain 564 disease targets (Figure 2A). The GSE10072 dataset was corrected (Figure 2B) for differentially expressed gene analysis, and 1624 DEGs were obtained, including 750 up-regulated genes and 874 down-regulated genes. Figure 2C,D displays the heatmap and volcano diagram of differentially expressed genes (DEGs). Seven marker genes were obtained by taking the intersection of DEGs, OSU-2S targets, and disease genes (Figure 3A,B).

### 2.2. Survival and Expression Analysis of Marker Genes

**Figure 4:**
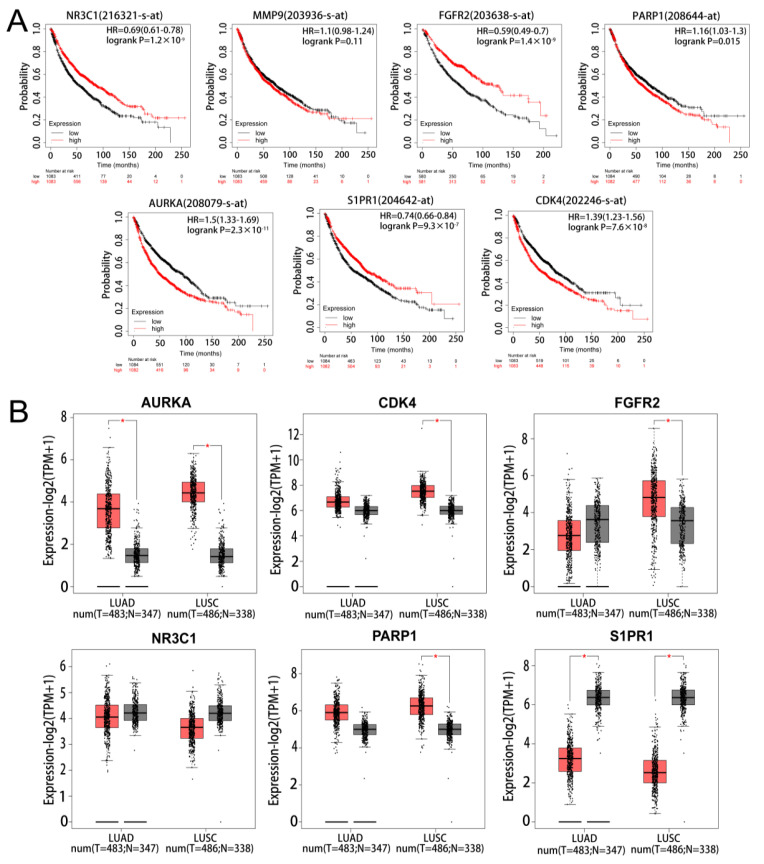
Survival and expression analysis of marker gene. (**A**) KM survival analysis of marker gene. (**B**) Expression analysis of marker in lung adenocarcinoma and lung squamous carcinoma, light red represents tumour and grey represents normal. * represents *p* < 0.05.

The results of survival analysis of the marker genes found that 6 genes (*PARP1*, *CDK4*, *FGFR2*, *AURKA*, *NR3C1*, and *S1PR1*) had significant prognostic significance in NSCLC. However, *MMP9* was not statistically significant in the prognostic analysis of NSCLC (Figure 4A). The results of the mRNA expression analysis of the marker genes in LUAD and LUSC found *AURKA* to be significantly up-regulated and *S1PR1* to be significantly down-regulated in LUAD and LUSC (Figure 4B). On the basis of the results of the two analyses, *AURKA* and *S1PR1* were chosen as hub genes.

### 2.3. Functional Enrichment of Hub Genes

**Figure 5:**
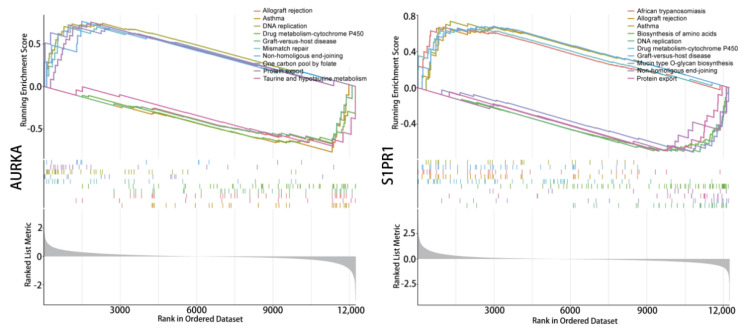
Single-gene signaling pathway analysis. Single gene GSEA-KEGG pathway enrichment analysis of *AURKA* and *S1PR1*.

Enrichment analysis of the single-gene GSEA-KEGG pathway (Figure 5) found that *AURKA* and *S1PR1* are all involved in DNA replication signals and can affect the cytochrome P450 enzyme system in drug metabolism, which suggests that hub genes affect tumour progression during the DNA replication phase of tumour cell growth while also having the potential for involvement in tumour drug resistance. In addition, *AURKA* are involved in the functional pathway of the one-carbon unit donor folate, *S1PR1* directly affects protein output, and *S1PR1* plays roles in amino acid synthesis. These results suggest that hub genes could be involved in multiple key signalling mechanisms in tumour growth and may be crucial in tumour drug resistance.

### 2.4. OSU-2S Effectively Inhibited the Cell Viability, Migration, and Colony Formation of A549 Cells

**Figure 6:**
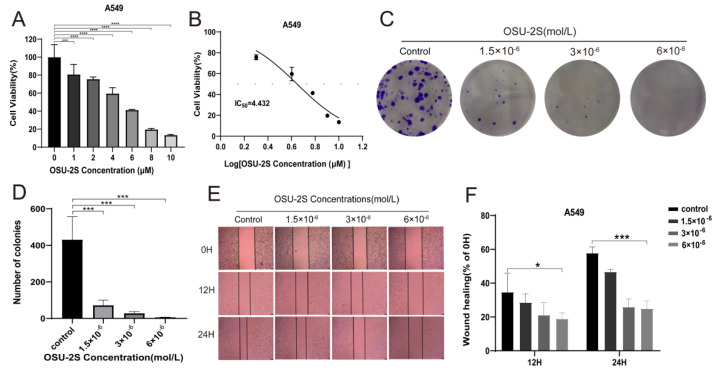
A549 cells were treated with OSU-2S for CCK-8, clone formation assays, and wound-healing assays. (**A**,**B**) A549 cells were treated with different concentrations OSU-2S for 24 h before the cell viability was determined via CCK8. (**C**,**D**) Crystalline violet staining to analyze colony formation in A549 cells treated by three different concentrations (1.5 × 10^−6^, 3 × 10^−6^ and 6 × 10^−6^ mol/L) of OSU-2S. All values are expressed as the mean ± SD (n = 3). (**E**) A549 cells were treated with three different concentrations (1.5 × 10^−6^, 3 × 10^−6^ and 6 × 10^−6^ mol/L) OSU-2S for 24 h, and the intercellular spacing was observed at different time points (0 h, 12 h, and 24 h). (**F**) The statistical analyses of the migration ability. All values are expressed as the mean ± SD (n = 3). Scale bar, 50 μm. * represents *p* < 0.05, *** represents *p* < 0.001 and **** represents *p* < 0.0001.

To verify the effect of OSU-2S on lung adenocarcinoma cells during growth, A549 cells were selected for administration by different concentrations of OSU-2S for 24 h. The CCK-8 results showed OSU-2S to have the ability to inhibit A549 cell proliferation (Figure 6A,B). Based on the CCK-8 assay results, we confirmed by clone formation and wound-healing assay experiments that OSU-2S-treated A549 cells exhibited lower migration and clone formation ability (Figure 6C,F).

### 2.5. Validation of AURKA, S1PR1 Protein Expression

**Figure 7:**
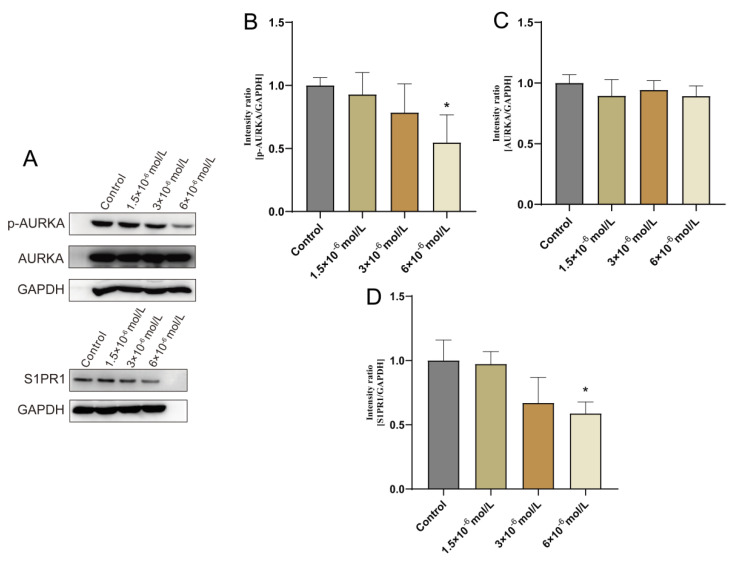
Effect of OSU-2S on the expression of *AURKA*, *S1PR1* detected by western blotting assay A549 cells were exposed to three different concentrations (1.5 × 10^−6^, 3 × 10^−6^, and 6 × 10^−6^) of OSU-2S for 24 h. (**A**) Effect of different concentrations of OSU-2S on the expression of *AURKA* and *S1PR1* protein. (**B**–**D**) The statistical analyses of the protein expression. All values are expressed as the mean  ±  SD (n = 3). * represents *p* < 0.05.

To confirm OSU-2S’s impact on lung adenocarcinoma cells during growth, A549 cells were selected for administration by different concentrations of OSU-2S for 24 h. The results showed that OSU-2S has the ability to inhibit A549 cell proliferation in a dose-dependent manner. Furthermore, the effect of OSU-2S on the differentially expressed proteins *AURKA*, *p-AURKA*, and *S1PR1* was further verified by Western blotting. The results showed that different OSU-2S concentrations (1.5 × 10^−6^, 3 × 10^−6^, and 6 × 10^−6^ mol/L) could significantly inhibit the *p-AURKA* and *S1PR1* expressions with the increase in the concentration after treating A549 cells for 24 h, as can be seen in Figure 7A–D. These results confirmed that OSU-2S had a significant regulatory effect on *p-AURKA* and *S1PR1* expressions in A549 lung adenocarcinoma cells, and the effect on *AURKA* expression was not obvious. However, the specific signalling pathways that are affected by the regulation of *p-AURKA* and *S1PR1* expressions require further in-depth investigation, which our next study will focus on.

### 2.6. OSU-2S Showed Anti-Tumour Activity in a Mouse Lung Cancer Model

**Figure 8:**
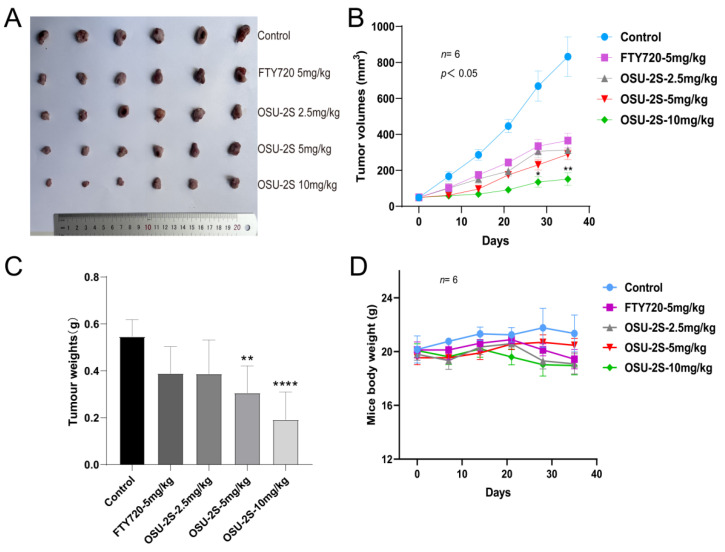
Anti-tumour effect of OSU-2S on A549 xenograft lung cancer mice. (**A**) Tumours were isolated subcutaneously from mice after 35 days of treatment (n = 6). (**B**) Tumour volume was measured weekly during the experiment (n = 6). (**C**) Tumour weights were measured after 35 days of treatment (n = 6). (**D**) Weights of mice were measured weekly (n = 6) during treatment with β-hydroxypropyl-cyclodextrin, FTY720, and OSU-2S (2.5 mg/kg, 5 mg/kg, 10 mg/kg). * represents *p* < 0.05, ** represents *p* < 0.01 and **** represents *p* < 0.0001.

An A549 xenograft lung cancer mouse model was used for evaluating the anti-tumour effect of OSU-2S. A549 cells were injected into the axilla of mice as a means of producing primary tumours at the injection site. Different doses of OSU-2S were then administered when the tumour volume reached 50 mm^3^. The tumours were isolated subcutaneously from the mice after 35 days of treatment and then weighed (n = 6). In comparison to the solvent (β-hydroxypropyl-cyclodextrin), the tumour volume and total weight were considerably reduced by OSU-2S at 5 and 10 mg/kg (Figure 8A–C). Additionally, the mice’s weight did not significantly decrease (Figure 8D), which indicated that OSU-2S at the dosage of 10 mg/kg is well tolerated in BALB/c nude mice.

### 2.7. Immunohistochemical Detection of Vascular Endothelial Growth Factor and Tumor Proliferation Markers

**Figure 9:**
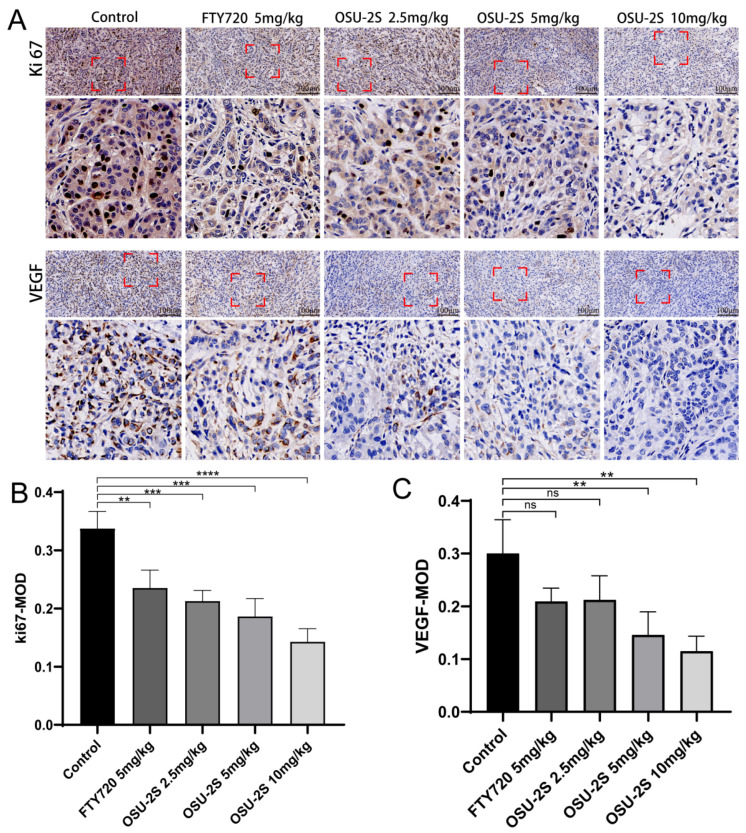
The effect of OSU-2S on the expression of proliferation and apoptosis markers in tumour tissues. (**A**) Representative IHC images of VEGF and Ki67 of the tumors. (**B**,**C**) Quantification analysis of immunoreactive cells for VEGF and Ki67. Data are presented as mean  ±  SD (n  =  3). Scale bar, 50 μm. ** represents *p* < 0.01, *** represents *p* < 0.001 and **** represents *p* < 0.0001. ns not significant.

Vascular endothelial growth factor (VEGF) expression is closely associated with angiogenesis in tumour tissues and lung cancer growth. Increased VEGF uexpression can increase tumour tissue angiogenesis, promoting lung cancer growth. Therefore, the determination of VEGF expression using immunohistochemical methods and the determination of tumorigenicity based on proliferation markers (Ki-67) are essential for the detection of the anti-tumorigenic capacity of drugs in vivo. OSU-2S significantly downregulated the expression levels of Ki-67 and VEGF in tumour tissues and inhibited tumour growth (Figure 9A–C). The results suggest that OSU-2S has a definite inhibiting effect on the development of lung cancer in vivo.

## 3. Discussion

The most prevalent kind of NSCLC is LUAD [17], and it develops abnormally from type II alveolar cells of the airway epithelium that secrete mucus and other substances [2]. It is the main culprit responsible for mortality linked to lung cancer in individuals. In contrast to other lung cancer subtypes, LUAD exhibits a gradual progression and lacks apparent clinical signs during its initial stages. Approximately 30–75% of patients are already in an advanced phase when they receive diagnosis, by which time radical treatment is impossible [18]. Therefore, early detection and treatment of LUAD are crucial [19]. The exploration of potent early tumour indicators during routine examinations has emerged as a prominent aspect in LUAD investigations, and the discovery and application of drugs that target markers is also key to LUAD treatment. OSU-2S is a derivative of FTY720 that also has inhibitory effects on a variety of tumour cells, and following structural modification, it was found that OSU-2S was not phosphorylated, thereby avoiding the immunosuppressive effect of FTY720 [13,14]. In the current era of increasing cancer disease, the new anticancer drug OSU-2S is highly expected in tumour therapy, but current basic research on the anticancer effect of OSU-2S is lacking and this is an urgent problem that requires solving. Therefore, this study utilized network pharmacology and bioinformatics methods to obtain potential targets for the treatment of NSCLC by OSU-2S, verified the efficacy of OSU-2S on NSCLC and its regulatory effects on potential targets through in vivo and in vitro experiments, and provided a reliable theoretical foundation for the early detection and treatment of LUAD.

In this study, two genes (*AURKA* and *S1PR1*) were screened as hub genes of OSU-2S treatment NSCLC by survival analysis and expression analysis, and the study has confirmed the tumour-growth-suppressive effect of OSU-2S in in vivo and in vitro experiments.

*AURKA*, *AURKB*, and *AURKC* are the three members of the serine-threonine kinase family. *AURKA* and *AURKB* play essential roles in cell division, proliferation, and migration through the regulation of the cell mitosis process [20]. A related study found that *AURKA* acts as an oncogene to facilitate the promotion of the growth of many tumour types, such as solid tumours and haematologic malignancies [21,22,23]. A related study also found that knockdown *AURKA* substantially escalated the effectiveness of radiation on HCT116 and HT-29 cell proliferation. The combined impact of *AURKA* inhibition and radiation demonstrated a potent capacity to suppress cellular migration and metastasis while also instigating apoptosis synergistically [24]. Relevant bioinformatics analysis suggests *AURKA* may be a key gene in lung cancer, which could lead to poor prognosis [25]. Furthermore, *CXCL5*, which is a key downstream of *AURKA*, was demonstrated to be suppressed after *AURKA* silencing in NSCLC. The *AURKA-CXCL5* axis is critical to regulate the radiosensitivity of NSCLC in the cell autophagy process [26]. Bioinformatics analysis in this study highlighted the enhanced expression of *AURKA* in LUAD tissues, which was consistent with the above findings. The OSU-2S treatment of A549 cells resulted in minimal changes in total *AURKA* protein, while the expression of *p-AURKA* appeared to be significantly reduced in comparison to the controls. The above results suggest that *p-AURKA* is potentially a key marker for LUAD growth and metastasis, and it may also be a potential target of OSU-2S for LUAD treatment.

*S1PR1* (Sphingosine-1-phosphate receptor 1) is a G protein-coupled receptor that is responsible for the mediation of the effects of the biologically active sphingosine 1-phosphate (S1P). *S1PR1* is a member of a subfamily of receptors known as sphingosine-1-phosphate receptors. These receptors are crucial in the maintenance of the structural integrity of endothelial cells, facilitating cell migration, modulating immune responses, and promoting angiogenesis [27]. Related studies have found S1PR1 to exhibit pronounced expression in malignant bladder cells, which correlates with an unfavourable prognosis among affected individuals [28]. S1P was also found to be able to enhance cancer cell viability and promote cancer cell growth and metastasis by binding to *S1PR1* [29]. Furthermore, according to related research, *S1PR1* regulates lymphocyte proliferation and differentiation inside the cancer microenvironment [30]. It was also reported that genetic deletion of S1P receptor 1 (*S1PR1*) in macrophage-infiltrated mouse mammary tumours prevents lung metastasis and tumour lymphangiogenesis [31]. In lung-cancer-related studies, it was found that the proliferation and invasion of NSCLC cells in vitro and tumor growth in vivo could be promoted by up-regulating the expression of *S1PR1*. In addition, inhibition of *S1PR1* can be achieved by targeting inhibited NSCLC cell proliferation, migration, and invasion, while inducing more NSCLC cell apoptosis [32,33]. We identified *S1PR1* as an anticancer target with potential research value in LUAD by network pharmacology. It was also further confirmed that OSU-2S can down-regulate the expression of *S1PR1* in A549 cells in protein immunoblotting experiments. However, the specific signalling pathway through which OSU-2S affects *S1PR1* requires further investigation.

Western blotting analysis confirmed that OSU-2S could down-regulate *p-AURKA* and *S1PR1* expressions in A549 cells. GSEA analysis showed *AURKA* and *S1PR1* to be involved in various important biological cell growth processes, including one-carbon unit donor folate function, DNA replication signalling, and amino acid synthesis, which may be the key to the anticancer effect of OSU-2S. Studies on anticancer drugs have demonstrated that methotrexate interferes with folate metabolism as a means of blocking the utilisation of one carbon unit in tumour cells, which inhibits cell division. Cisplatin is a common drug used to treat non-small-cell lung cancer, and its mechanism of action is to stop the DNA replication of tumour cells and block cell division to produce an anti-tumour effect [34,35,36]

## 4. Materials and Methods

### 4.1. Data Sources

The chemical structure of OSU-2S was obtained from the PharmMapper (http://www.lilab-ecust.cn/pharmmapper/, accessed on 10 October 2022) database for identifying its target. The targets of OSU-2S were searched in the Swiss TargetPrediction (http://www.swisstargetprediction.ch/, accessed on 10 October 2022) database using Canonical SMILES of OSU-2S as the keyword. (The number of OSU-2S targets obtained was small, so targets were not screened based on relevant conditions.) The R GEO query package [37] was used for obtaining the original dataset GSE10072 from the high-throughput Gene Expression Omnibus, (GEO) (https://www.ncbi.nlm.nih.gov/geo/, accessed on 23 July 2023) database in the GPL96 ([HG-U133A] Affymetrix Human Genome U133A Array) platform as the experimental set for analysis (including 58 lung adenocarcinoma tissues and 49 normal lung tissues). To better match the lung adenocarcinoma genes in the GSE10072 dataset, disease databases at Genecard (https://www.genecards.org/, accessed on 2 August 2023) and TTD (http://db.idrblab.net/ttd/, accessed on 20 August 2023) were searched for disease targets with “lung adenocarcinoma” used as the keyword (gene screening conditions were greater than or equal to the 4-fold median of correlation score).

### 4.2. Screening of Disease Genes and Differentially Expressed Genes

Disease genes obtained from the Genecard and TTD databases were merged, and duplicate values were removed. Before being filtered using the cytoHubba plugin in Cytoscape_v3.7.2, taking the intersection of the genes ranked in the top 700 (the genes ranked higher in the cytoHubba plug-in make more significance) in the four modules of Betweenness, Closeness, Degree, and Stress. The intersection of the top 700 genes was defined as the disease genes. The dataset that was used in this study was corrected by NormalizeBetweenArrays to ensure the removal of the batch effect, and the differentially expressed genes (DEGs) between healthy lung tissues and adenocarcinoma tissues was examined employing the R software (Version 4.3.1) limma package [38] with |log2FC| > 0.5 and *p* < 0.05 as the criteria to determine DEGs. The criteria of log2FC > 0.5 and *p* < 0.05 were used to identify differentially expressed genes (DEGs). The intersecting genes of OSU-2S targets, disease genes, and DEGs were obtained as marker genes for OSU-2S inhibition of NSCLC by Using the jvenn diagram tool (https://jvenn.toulouse.inra.fr/app/example.html, accessed on 20 August 2023) and Microsoft Excel.

### 4.3. Survival Analysis and Expression Analysis of Marker Genes

The marker gene survival analysis was carried out employing the KM Plotter database (http://kmplot.com/analysis/, accessed on 19 September 2023). Employing the GEPIA2 database, expression analysis of the marker genes in lung adenocarcinoma and lung squamous carcinoma was carried out (http://gepia2.cancer-pku.cn/, accessed on 19 September 2023). The results of the two analyses were integrated, and whether the *p*-value was significantly different was used as a screening condition for hub genes (*p* < 0.05 was considered to be significantly different).

### 4.4. GSEA-KEGG Enrichment and Protein Expression Analysis of Hub Genes

Correlations between hub genes and other genes in the GSE10072 dataset were analysed by the R software GSEA package. The KEGG signalling pathway set was then called a predefined set for the detection of the enrichment of hub genes in the signalling pathway set.

### 4.5. Cell Experiments

#### 4.5.1. Cell Culture

Lung adenocarcinoma A549 cell lines (American Type Culture Collection(ATCC), Manassas, VA, USA) were cultured in F12K (Procell, Wuhan, China) medium supplemented with 100 mg/mL streptomycin (TRANS, Beijing, China) and 100 U/mL penicillin (TRANS, Beijing, China), 10% fetal bovine serum (FBS) (Sigma-Aldrich, Burlington, MA, USA), under 37 °C, 5% CO_2_ and high humidity in a constant temperature incubator.

#### 4.5.2. Cell Counting Kit (CCK)-8 Assay

The Cell Counting Kit-8 (CCK-8) (Solarbio, Beijing, China) was used to evaluate the cell viability according to the manufacturer’s instructions (Bioss, Beijing, China). Cell culture conditions were as described in Section 4.5.1, and A549 cells (3 × 10^3^ cells/well) were incubated in 96-well plates for 24 h. The CCK-8 solution (10 μL) was added to each well, and the plates were incubated for 1 h at 37 °C; then the absorbance at 450 nm wavelength (OD 450) was measured in a microplate reader (Biorad, Hercules, CA, USA).

#### 4.5.3. Cell Migration

Cell culture conditions were as described in Section 4.5.1, and well-grown A549 cells were inoculated into 6-well plates at 1 × 10^−6^ cells per well and placed in an incubator at 37 °C, 5% CO_2_ for 24 h in order to form a monolayer of cells the following day. The tip of a 1000 μL micropipette was used for scratching the monolayer of cells to ensure that a uniform scratch was formed in the monolayer. The cells were rinsed as a means of removing cellular debris and incubated in 1% FBS medium with or without different concentrations of OSU-2S (1.5 × 10^−6^, 3 × 10^−6^ and 6 × 10^−6^ mol/L) for 24 h. Images were taken of the wells every 6 h using light microscope observation. All the data were analysed using Image-J software((Version 1.8.0).

#### 4.5.4. Colony Formation Assay

An in vitro clone formation assay was used for measuring colony formation. Cell culture conditions were as described in Section 4.5.1, and A density of 200 cells per well was inoculated into a 6-well plate and placed in an incubator at 37 °C with 5% CO_2_ for overnight incubation in order to ensure adhesion. The cells were incubated in the medium with or without different concentrations of OSU-2S (1.5 × 10^−6^, 3 × 10^−6^, and 6 × 10^−6^ mol/L) the following day for 14 days. Following the conclusion of the incubation period, the cells were washed twice with PBS (Procell, Wuhan, China). Four percent paraformaldehyde solution was used for fixation for 30 min, and the cells were stained with crystal violet dye for 10 min at room temperature. The cells were then washed using deionised water and were allowed to dry overnight before the number of different clones in each condition were counted. The experiment was repeated three times.

#### 4.5.5. Western Blotting

Cell culture conditions were as described in Section 4.5.1, and 6-well plates were used to seed A549 cells (5 × 10^5^ cells/well). The cells were incubated for 12 h before being exposed to OSU-2S for 24 h. Following cell harvesting, entire cells were lysed in RIPA solution in order to extract all of the proteins. Before being transferred to a polyvinylidene fluoride membrane (PVDF; Millipore, Boston, MA, USA) sodium dodecyl sulphate-polyacrylamide gel electrophoresis. After that, the membrane was washed with 1×TBST (Solarbio, Beijing, China) solution for 3 times, 5 min each time. The membranes were sealed for an hour at room temperature using 5% skim milk. The membrane was subjected to an overnight 4 °C incubation with the primary antibody (anti-AURKA, anti-S1PR1 at 1:1000; Affinity, San Francisco, CA, USA), and the membrane was washed with 1×TBST solution for 3 times, 5 min each time. An hour-long room temperature incubation was carried out with the horseradish peroxidase-conjugated secondary antibody (Affinity, San Francisco, CA, USA), and the membrane was washed with 1×TBST solution for 3 times, 5 min each time. Bound proteins were visualized using a chemiluminescence imaging system (Biorad, Hercules, CA, USA).

### 4.6. Animal Experimentation

#### 4.6.1. Experimental Mouse

Male BALB/c nude mice (4-6 weeks old, body weight 20 ± 2 g) were purchased from Charles River and placed in a controlled experimental environment conforming to spf grade standards in the Animal Experimentation Center of Xinjiang Medical University, at a temperature of 23 ± 2 °C, with standard food and sterile drinking water, and a light/dark cycle of 12 h. The present study was approved by the Ethics Committee for Experimental Animals of Xinjiang Medical University (Ethics The study was approved by the Laboratory Animal Ethics Committee of Xinjiang Medical University (Ethics Approval No. IACUC-JT-20230423-20).

#### 4.6.2. Establishment of a Xenograft Mouse Model and Grouping

After the mice were acclimatized, well-conditioned A549 cells (1 × 10^7^) were injected subcutaneously into the axilla. When the tumour diameter reached 50 mm^3^, the mice were randomly assigned to five groups (n = 6) and given intraperitoneal injections of FTY720 (5 mg/kg), OSU-2S (2.5 mg/kg, 5 mg/kg, and 10 mg/kg groups) and β-hydroxypropyl-cyclodextrin as a control once every other day for 35 days. The growth of the tumour and the body weight of the mice was recorded on a weekly basis. The formula used to determine the tumour volume was as follows: volume = length × width^2^ × 1/2.

#### 4.6.3. Immunohistochemistry of Tumour Tissue

The mice’s tumour tissues were fixed for 24 h with a 4% para-formaldehyde solution (Solarbio, Beijing, China) after being cleaned with saline. They were then dehydrated and embedded in paraffin wax. The slices were baked at 60 °C for 12 h, placed in a slice box, and stored in the refrigerator at 4 °C for future use. The sections were dewaxed and then incubated with goat serum and an endogenous peroxidase blocker. After that, the slices were incubated with anti-Ki67 (Affinity, San Francisco, CA, USA) and anti-VEGF (Affinity, San Francisco, CA, USA) antibodies for an entire night at 4 °C. After incubating the sections with DAB and a goat anti-rabbit secondary antibody (Affinity, San Francisco, CA, USA), the slices were examined and photographed under a microscope.

### 4.7. Statistical Analysis

R 4.3.1 was utilised to conduct the processing and analysis of the data in the bioinformatics portion of the study, and the Wilcoxon rank sum test was employed to compare the two groups. The GraphPad Prism 8.0 (comparisons between groups were performed using one-way analysis of variance (ANOVA)) and ImageJ software were used for the statistical analysis of CCK-8, Western blotting, and immunohistochemistry results. All the experiments in this study were independently repeated a minimum of three times: *p* < 0.05 represents significant difference.

## 5. Conclusions

In conclusion, our study provides strong experimental evidence that OSU-2S has a significant anti-tumour effect in vitro and in vivo. Two hub genes (*AURKA* and *S1PR1*) were screened as early markers of LUAD pathogenesis and potential targets for OSU-2S to exert anti-LUAD by network pharmacology and bioinformatics methods. It was found that OSU-2S may down-regulate the expression of *S1PR1* and *AURKA*, which may prevent the growth of cancer cells. However, the exact molecular mechanism of action requires more thorough in vitro and in vivo experiments to be properly determined. The next study will focus on the molecular pathways that are involved in *AURKA* and *S1PR1* as a means of providing more feasible options for LUAD treatment.

## Data Availability

The datasets (GSE10072, GPL96) for this study can be found in the [The Gene Expression Omnibus (GEO) database]. All the data in this paper support the results of this study; other datasets used and/or analyzed during the current study are available from the corresponding author on reasonable request.
